# A survey of Canadian regulated complementary and alternative medicine schools about research, evidence-based health care and interprofessional training, as well as continuing education

**DOI:** 10.1186/1472-6882-13-374

**Published:** 2013-12-28

**Authors:** Karine Toupin April, Isabelle Gaboury

**Affiliations:** 1 Department of Epidemiology and Community Medicine, Faculty of Medicine, University of Ottawa, Ottawa, Ontario, Canada; 2 Centre for Global Health, Institute of Population Health, University of Ottawa, Ottawa, Ontario, Canada; 3 Children’s Hospital of Eastern Ontario Research Institute, Ottawa, Ontario, Canada; 4 Department of Pediatrics, Faculty of Medicine, University of Ottawa, Ottawa, Ontario, Canada; 5 Department of Family Medicine, Université de Sherbrooke, Sherbrooke, Quebec, Canada; 6 Centre de recherche clinique Étienne-Lebel, Centre Hospitalier Universitaire de Sherbrooke, Sherbrooke, Quebec, Canada

**Keywords:** Complementary and alternative medicine, Continuing education, Curriculum development, Evidence-based health care training, Interprofessional training, Research training

## Abstract

**Background:**

While some effort has been made to integrate complementary and alternative medicine (CAM) information in conventional biomedical training, it is unclear whether regulated Canadian CAM schools’ students are exposed to research activities and continuing education, or whether topics such as evidence-based health care and interprofessional collaboration (IPC) are covered during their training. Since these areas are valued by the biomedical training field, this may help to bridge the attitudinal and communication gaps between these different practices. The aim of this study was to describe the training offered in these areas and gather the perceptions of curriculum/program directors in regulated Canadian CAM schools.

**Methods:**

A two-phase study consisting of an electronic survey and subsequent semi-structured telephone interviews was conducted with curriculum/program (C/P) directors in regulated Canadian CAM schools. Questions assessed the extent of the research, evidence-based health care, IPC training and continuing education, as well as the C/P directors’ perceptions about the training. Descriptive statistics were used to describe the schools’, curriculum’s and the C/P directors’ characteristics. Content analysis was conducted on the interview material.

**Results:**

Twenty-eight C/P directors replied to the electronic survey and 11 participated in the interviews, representing chiropractic, naturopathy, acupuncture and massage therapy schools. Canadian regulated CAM schools offered research and evidence-based health care training as well as opportunities for collaboration with biomedical peers and continuing education to a various extent (58% to 91%). Although directors were generally satisfied with the training offered at their school, they expressed a desire for improvements. They felt future CAM providers should understand research findings and be able to rely on high quality research and to communicate with conventional care providers as well as to engage in continuing education. Limited length of the curriculum was one of the barriers to such improvements.

**Conclusions:**

These findings seem to reinforce the directors’ interest and the importance of integrating these topics in order to ensure best CAM practices and improve communication between CAM and conventional providers.

## Background

Scientific literature indicates an increased interest in complementary and alternative medicine (CAM) among the general public and a tendency towards increased use, especially in patients with chronic diseases [1-3]. Most individuals who use CAM also consult conventional care providers [4]. This highlights the importance of both conventional and complementary professionals understanding one another’s practices and paradigm, in order to encourage a fruitful communication process, and promote an integrated vision of health care [5]. However, communication and collaboration between these health care providers is often not optimal [6]. Conventional and complementary health care providers are occasionally at diametrically opposite ends of the health care spectrum in their beliefs and practices, and use different terminology to communicate information, which may create misunderstandings and confusion for their patients [5].

Some effort has been made to integrate CAM information in conventional biomedical training and practice [3,7-9], which may help future conventional care providers to understand CAM approaches. A CAM curriculum presenting practical and evidence-based information about CAM is available in a few medical schools throughout North America [10]. In Canada, many medical schools include CAM in their curriculum, but few schools are offering CAM training in a formalized manner [11].

In parallel, the integration of some research, evidence-based health care and interprofessional collaboration (IPC) training, as well as some evidence-based continuing education in CAM schools, may help to bridge the gap between conventional care and CAM. In fact, such training may help CAM practitioners to identify, appreciate, and generate scientific evidence in order to improve treatment [12,13], as well as develop a common language with biomedical providers. However, it is unclear whether Canadian CAM schools’ students learn about topics valued by biomedical providers such as the conduct, interpretation and clinical implications of research. Furthermore, the extent of training in IPC with biomedical providers offered to CAM students is unknown. Limited evidence derived from a search of information posted on the Canadian regulated CAM schools’ websites suggested that some CAM schools have integrated these competencies into their curriculum with varying levels of success [14]. However, since the comprehensiveness of those websites was highly variable, the research and evidence-based content of the future Canadian CAM practitioners’ curriculum remained to be rigorously assessed.

To address this gap in the literature, our research team conducted a two-phase study comprised of an electronic survey and subsequent telephone interviews targeting curriculum/program (C/P) directors of Canadian regulated CAM schools. The aim of this study was to describe the research and evidence-based health care training, as well as IPC and continuing education, offered at these schools. The ultimate goal of this paper was to assess and describe a knowledge gap in the Canadian regulated CAM schools, which, if addressed, could encourage collaborative attitudes and practice, as well as communication, between future biomedical and CAM healthcare practitioners.

## Methods

### Population

#### Phase 1

Canadian regulated CAM schools (based on specific provincial regulations) were identified using the CAMline website and regulating bodies’ websites. The main C/P director or person responsible for curriculum development was identified at each Canadian regulated CAM school through the school’s website or by contacting the school. Regulated schools included two chiropractic colleges, two naturopathic colleges, 19 acupuncture/traditional Chinese medicine colleges as well as 28 massage therapy colleges.

#### Phase 2

The second phase of the project consisted of a subsample of respondents to the survey who volunteered to participate in a telephone interview.

Ethics approval was obtained from the Ottawa Hospital Research Ethics Board (OHREB) for the duration of the study (March 2010-March 2011). C/P directors who completed the survey and volunteered for the interviews were considered to have given their consent if they answered the survey and contacted the research team to be interviewed. C/P directors who participated in the interviews also gave their verbal consent, which was noted by the researchers in the study documents.

### Data collection

#### Phase 1

An electronic survey was sent to all identified Canadian regulated CAM schools’ C/P directors via Survey Monkey. An introductory email was sent by the research team to all eligible participants to invite them to participate in a survey. Reminders were sent after one and two weeks to ensure maximum participation. A mailed copy of the survey was also sent to C/P directors who had not responded after two reminders.

#### Phase 2

A single semi-structured telephone interview was conducted by the main author (KTA) with each participant from a subsample of regulated Canadian CAM school C/P directors in order to gather participants’ perceptions of their curriculum in more depth. The main author is a female researcher, an occupational therapist by training with a doctorate in public health and epidemiology, and training in both qualitative and quantitative research. An interview guide and probes were used to clarify perceptions. The interview questions were *a priori* tested with 2 CAM researchers to ensure clarity. The interview was designed to last between 15 to 30 minutes. An interview information letter was sent by email and the interviews were scheduled for those who agreed to participate. Two reminders were sent.

### Tools

#### Phase 1

The survey (see Additional file 1) was developed using a questionnaire similar to the one used for the “CAM in UME” (Undergraduate Medical Education) project, which assessed the students’ and directors’ views of CAM training offered in undergraduate medical programs [15]. The questionnaire was *a priori* pilot tested with three CAM school C/P directors and researchers to ensure participants’ understanding and ease of use. The questionnaire was modified according to the comments received and no data was analyzed from this methodological exercise. The goal of the survey was to evaluate the extent of research training, evidence-based health care training and opportunities for continuing education and collaboration with biomedical peers offered at these schools. First, characteristics of the schools (e.g. number of educators, number of students), curricula (length, CAM specialty) and C/P directors (e.g. professional training) were assessed. Then, the extent of training in the areas of interest was evaluated using the number of hours that they were taught during the program. The survey assessed the pedagogical approaches used to teach these topics (such as readings, lectures, invited guests, case studies, research projects, internships). The survey also measured C/P directors’ satisfaction of the training offered using a five-point Likert scale ranging from very dissatisfied to very satisfied, as well as the changes that were anticipated for the next two to five years, and improvements they felt necessary for an eventual curriculum renewal. Finally, directors were asked about barriers to such changes, as well as the support that they would receive from the students and the administration (rated on a five-point Likert scale ranging from very unlikely to very likely).

#### Phase 2

The interview guide for the telephone interviews was developed *de novo* by the research team (see Sample of questions asked in the telephone interviews section). The guide was *a priori* tested with 2 CAM researchers to ensure clarity. The interview questions did not require any modifications and the data from those pilot interviews was excluded from the analysis. The goal of these questions was to understand directors’ perceptions of the training offered at their school in more depth. The interviewer asked about the extent of the training offered, as well as the C/P directors’ satisfaction, perceived need for improvement and perceived barriers to such changes. The interviewer kept a written account of the interview.

### Sample of questions asked in the telephone interviews

1. Which CAM accredited program(s) do you offer?

2. Review of their answers to the survey:

a) Could you describe the continuing education offered in your CAM program(s)?

b) Could you describe the research training offered in your CAM program(s)?

c) Could you describe the training about scientific proofs about the efficacy and safety of treatments?

d) Could you describe opportunities for CAM professionals to interact with biomedical peers (e.g. nurse, rehabilitation professional, medical doctor) in their practice?

3. Do you expect changes to be made to the curriculum concerning these areas?

4. Which changes would you make to the training offered in your program(s)?

5. What are the barriers to such changes?

6. How do you think these modifications would be perceived at your school?

### Data management and analysis

Results from the survey were imported into SPSS files to be analyzed quantitatively. Descriptive statistics were used to describe the characteristics of the schools, curricula and C/P directors. The level of satisfaction with the various types of training and the perceived support from students and the administration were described using a three-point Likert scale to facilitate the interpretation of results. Emergent content analysis was performed on the interview material, *i.e.*, themes were established following a preliminary examination of the data and discussion between the authors. The coding process was also guided by the following themes: the directors’ satisfaction, perceived need for improvement and perceived barriers to such changes, as well as changes that would be made to the curriculum in the next years. The coding was then conducted independently by KTA and issues were discussed with IG. As part of the process, the interviewer summarized the interview and asked participants to confirm her interpretation.

## Results

### Phase 1

Among the 51 potential participants, 28 replied to the electronic survey, but three did not complete most of the questions. Of the remaining 25 participants who answered most of the questions (see n for each result), one came from a chiropractic school, two from naturopathy schools, 10 from massage therapy schools, and eight from acupuncture schools (see Table 1 for characteristics of the schools). 16 respondents were managers, 17 were health professionals, including CAM providers (n = 15), a medical doctor, other biomedical practitioners (e.g. nurse, rehabilitation professional) (n = 4) and a researcher (response rate = 88%). Respondents had been trained in Canada (n = 14), the United States (n = 3), Asia (n = 5) or Europe (n = 1).

**Table 1:** Characteristics of the CAM schools

**Number of CAM schools:**	
Discipline:	
Chiropractic	1
Naturopathy	2
Massage	10
Acupuncture	8
Size of the schools:	
Number of students in schools:	
<50	8
51-100	5
>100	8
Number of educators in schools:	
<10	6
11-20	10
21-50	3
>50	2
Educators’ training:	
Holding a doctorate (PhD)	6
Holding a medical degree (MD)	8

According to schools’ websites, overall training length ranged from 1,930 to 5,000 hours, naturopathic and chiropractic schools offering the most (mean = 4,600 hours) and massage therapy (mean = 2,270 hours) and acupuncture (mean = 2,167 hours) following. Research methods and evidence-based health care training, as well as opportunities for collaboration with biomedical peers and continuing education, were offered to a variable extent (see Table 2). They were offered by 81%, 91%, 80%, and 58% of the schools respectively. These statistics are detailed in the following paragraph.

**Table 2:** Training offered in Canadian regulated CAM schools

	**Number of schools offering each range of training**							
Type of training	<35 hours	36-70	71-105	106-140	141-175	176-210	>210	Missing data*
Research methods	10	5	0	0	0	0	0	2
Evidence-based	2	3	2	0	0	0	10	2
health care								
Interprofessional	4	5	0	0	0	1	4	2
Training								
Continuous	3	2	1	2	0	0	3	3
Education								

*Two respondents did not provide the number of hours of training for research methods, evidence-based health care and interprofessional training, and three respondents did not provide this information for continuing education.

According to the survey, a total of 12 (63.16%) schools employed educators who were regularly involved in research. Also, 17 schools offered some research methods training. Most offered one or two research methods courses (n = 14 schools) and/or a research project (n = 14), sometimes a research project involving recruitment and data collection, but most often a literature search. Courses often focused on research methodology and literacy, basic epidemiology and biostatistics. The websites of three schools mentioned the writing of a thesis. Most respondents were either satisfied (n = 8, 44%) or neutral (n = 7, 39%) with this training. Three respondents (17%) were dissatisfied with research methods training. Evidence-based health care training was offered in 19 schools, mostly in the form of lectures with readings (n = 16) and case studies (n = 13). Most of the respondents were satisfied (n = 12, 63%) or neutral (n = 6, 31%) with the training they offered in this respect. Only one respondent was dissatisfied with the evidence-based health care training offered. Opportunities for collaboration with biomedical peers and training about this topic were offered in 16 schools, mostly in the form of invited speakers (n = 8) and internships (n = 6). According to the schools’ websites, internships were mostly offered in a clinic at the school, and few schools offered internships in the conventional health care system. Most respondents were satisfied (n = 11, 58%) or neutral (n = 7, 37%), and one was dissatisfied with the opportunities and training they offered in this area. Continuing education was offered by 14 schools, mostly in the form of lectures with case studies and invited speakers. Results varied concerning the level of satisfaction, as six respondents were dissatisfied, six were neutral and seven were satisfied.

Many respondents (n = 16, 84%) mentioned that their school’s curriculum would be improved with respect to these areas in the next two to five years. Even though most C/P directors seemed satisfied, 63% still wished for some improvements in research conduct and continuing education. Both the administration of these schools as well as students were perceived to be supportive of these changes. However, some barriers to implementation included the difficulty in extending the length of the curriculum because of the associated financial burden. Other barriers included the lack of time to discuss possible curriculum improvements, and the lack of communication among the different schools, and between the regulating bodies and the schools about those changes. Finally, the lack of faculty knowledge and comfort with those issues, and the lack of rigorous research findings, would hinder changes.

### Phase 2

In the second phase of the project, 11 C/P directors participated in telephone interviews lasting from 11 to 51 minutes, with an average of 31 minutes per interview. Out of these, six were in charge of curriculum at massage schools, one at a naturopathy school, one at a chiropractic school, and three at acupuncture schools. Interview results confirmed survey results concerning training and further explained them. Respondents expressed a desire for future CAM providers to understand research findings in their field of practice, to be able to rely on high quality research, to be able to communicate with conventional care providers, as well as to engage in continuing education. The interviews revealed that research courses focused mostly on health research literacy and were designed to satisfy the requirements of their regulating bodies. Basic research methods and statistics were taught in order to ensure that students understood scientific proofs. Most C/P directors would like to offer more research training, as they felt it essential to understand the scientific literature and practice adequately. However, the lack of time in the curriculum precluded them from adding these courses. Respondents felt that students would not be willing to pay more for a longer curriculum involving more research, as students are often more interested in the clinical aspects of the profession than research.

Concerning the evidence-based health care training, many C/P directors said it was integrated throughout the curriculum. However, the lack of rigorous scientific proofs for many CAM treatments was an obstacle to evidence-based coursework and practice. In fact, respondents expressed the importance of research for the development of their profession and recognition by other health care providers. More research evidence on CAM would help the next generation of CAM providers to justify their treatments to both patients and other health care providers, which would improve their credibility both clinically and in research. Some C/P directors mentioned that their schools were offering graduate CAM training with the aim of developing researchers, and had witnessed an increasing interest from students.

Although CAM research was found to be important, interviews revealed that few educators conducted research on a regular basis (most being full-time clinicians), as they often lacked time and expertise to obtain funding and manage research projects. C/P directors expressed the desire to collaborate with academic researchers in order to obtain research funding and conduct high quality research.

Interviews showed that communication with other health care providers, including conventional ones, was discussed in most schools. Seven schools offered internships in the conventional care system or in the community where future CAM providers were able to interact with other professionals. This was true mostly for schools that were in collaboration with universities, as they could establish links to other health care settings more easily. Five C/P directors mentioned that conventional care providers were not necessarily open to CAM providers (mostly physicians), which made it difficult to collaborate with them and start internships in conventional settings. Respondents felt that schools should improve collaborations with health providers from other disciplines in order to offer more opportunities to their students.

Continuing education was not offered in many schools because of technical difficulties (e.g. lack of time and convenient location). C/P directors often felt it was not their main role, but mentioned that it is important for graduates to undertake continuing education on a regular basis to stay up to date in their practice. Continuing education usually involved clinical aspects of the profession and not research training.

Finally, C/P directors expressed the desire to have more time to discuss how their training could be improved, both with other school C/P directors and with their regulating bodies, with whom they do not necessarily agree on what students need.

## Discussion

Overall, results showed that many Canadian regulated CAM schools offered research methods and evidence-based health care training, as well as opportunities for collaboration with biomedical peers and continuing education, although to a various extent. This is reasonable given the various length of training for the various professions. Research training focused mostly on research literacy, which is the first step involved in using research findings to guide practice [12]. Evidence-based health care training and collaborations with biomedical peers were touched upon in the form of lectures and presentations. Barriers to these types of training included the difficulty in adding courses to the already full curriculum, the lack of scientific evidence in CAM, and the lack of recognition by other health care providers. Continuing education was offered less frequently by CAM schools than other types of training because of technical difficulties and the fact that they did not feel that it was their main responsibility.

Most respondents were neutral or satisfied (31% to 63%) with the training offered at their schools. However, improvements were felt to be important for the development of the profession and will be made in the next few years. This paradox may be explained by the fact that C/P directors acknowledged inherent barriers to training in these areas. The difficulty in extending the length of training, as well as the “marginality” of CAM compared to conventional care (possibly due to the difficulty in producing high quality evidence), constitute great challenges that will need to be addressed to improve training of future CAM providers.

Consistent with results found on the websites of the regulated CAM schools [14], research training length seemed to be related to curriculum length. Also, more educators at the schools with more lengthy curricula had conventional and CAM graduate or research degrees, and were involved in research on a regular basis, which may facilitate research and evidence-based training, as well as opportunities for IPC in conventional care settings for their students. It is possible that higher education in research creates links in the scientific community, which may help lead to research funding for educators’ and students’ projects. These educators may also serve as role models for their students, and influence their schools’ philosophy with respect to the importance of research and evidence-based education for practice. Furthermore, researchers with academic connections and collaborative opportunities in conventional care settings may be in a privileged position to advocate for the integration of CAM in the health care system. This is consistent with the desire of many C/P directors to have some of their educators and students involved in high quality research in order to improve the quality of care they provide, and facilitate the development and recognition of their profession. However, the difficulty in adding research training to the schools’ curricula has been addressed by the development of graduate CAM training programs, which may help to ensure the conduct of high quality research and evidence-based health care, ensuring the best treatments possible in practice [16].

### Limitations

Missing data in the survey precluded us from obtaining all relevant information on all types of CAM schools. We have tried to minimize this bias by taking into account results from our prior publication on the information posted on the CAM schools’ websites and the interviews. Triangulation of data helped to ensure the reliability of our results. Secondly, although very much in line with typical healthcare professional survey response rates [17], data should be interpreted with caution, considering the moderate response rate obtained. This is especially true for the acupuncture and massage schools, from which the lowest response rates were obtained. Finally, a social desirability bias may be present as interviewees may have wanted to please the researchers by mentioning their willingness to improve their curriculum.

## Conclusions

In the past, efforts have been focused mostly on integrating CAM information in conventional biomedical training and practice. This is not sufficient to ensure efficacious and satisfying collaborative relationships between future CAM and biomedical practitioners. Present findings seem to reinforce the C/P directors’ interest and the importance of integrating topics such as research methods, evidence-based health care and IPC training, as well as continuing education, into CAM providers’ training and practice in order to ensure best CAM practices and improve communication between CAM and biomedical providers. Although C/P directors were generally satisfied with their training, they acknowledged barriers that should be addressed to further improve the curriculum in these areas. Addressing the barriers precluding these improvements, such as the difficulty in modifying the content and length of the training, as well as the “marginality” of CAM compared to conventional care, may ultimately benefit the CAM professions and facilitate their recognition by the health community at large. The present article constitutes a road map that will help to identify and address the limitations with regards to the integration of research methods, evidence-based health care and IPC training, as well as continuing education to help to bridge the gap between conventional care and CAM.

## Abbreviations

CAM: Complementary and alternative medicine; C/P: Curriculum/program; IPC: Interprofessional collaboration; MD: Medical degree; PhD: Philosophy Doctor; UME: Undergraduate Medical Education.

## Competing interests

The authors declare that they have no competing interests.

## Authors’ contributions

KTA and IG have designed the study. KTA has collected the data and performed the analyses. KTA and IG interpreted the data, were involved in drafting the manuscript and approved the final version to be published. Both authors read and approved the final manuscript.

## Pre-publication history

The pre-publication history for this paper can be accessed here:

http://www.biomedcentral.com/1472-6882/13/374/prepub

## Supplementary Material

Additional file 1 CAM survey (Sent via survey monkey). Click here for file
