# Supplementary material for: A survey of Canadian regulated complementary and alternative medicine schools about research, evidence-based health care and interprofessional training, as well as continuing education
Source: BMC Complement Altern Med. 2013 Dec 28;13:374. doi: 10.1186/1472-6882-13-374 (PMC3877872; doi:10.1186/1472-6882-13-374)
Supplement: Additional file 1 — CAM survey (Sent via survey monkey). [file 1472-6882-13-374-S1.doc]

**Additional file 1**

CAM Survey

(Sent via Survey Monkey)

Dear administrators of CAM schools,

We are contacting you to obtain information on your CAM training program(s) and to ask your opinions about it. The overall aim of this research project is to describe the research and evidence-based health care research training, as well as opportunities for collaboration with biomedical peers, available in regulated Canadian complementary and alternative medicine (CAM) schools. To reach our objectives, we are sending an electronic questionnaire to administrators of regulated/accredited CAM schools in Canada. The questionnaire will take between **5 and 10 minutes** to complete. Your answers will be reported as part of a group, not individually. Therefore, you will not be identifiable in any reports or publications. All questionnaires will be coded with a unique identification code. All research related information will be kept confidential. All research related information will be kept for 15 years after completion of the study, and then destroyed. The Ottawa Hospital Research Ethics Board and the Ottawa Hospital Research Institute may review your study records for audit purposes. Your participation is voluntary. Completion of the survey will be considered as your implicit consent to participate in the current study.

The next step of this research project will be a short telephone interview of a few administrators to understand their perceptions of their curriculum in more depth. After completing the questionnaire, you will receive an email asking if you would like to participate in a 15 to 45 minutes telephone interview.

If you would like more information regarding this research project, please contact the investigators.

**Section 1: Describe yourself**

*1. Professional training (please check all that apply):*

❑ Medical doctor

❑ CAM practitioner, if yes, specify expertise: _________________________

❑ Other biomedical practitioner (e.g. nurse, rehabilitation professional)

❑ Researcher

❑ Manager (e.g. school director)

❑ Other, specify: _________________________

*2. Country of training:*

❑ Canada

❑ United States

❑ Other, specify: _________________________

**Section 2: Describe your CAM program(s)**

*1. In which year was/were your CAM program(s) founded?___________*

Program: _________________________________ Year:_______

Program: _________________________________ Year:_______

Program: _________________________________ Year:_______

Program: _________________________________ Year:_______

*2. How many students in total are enrolled in your CAM program(s)?*

❑ 1-25

❑ 26-50

❑ 51-75

❑ 76-100

❑ More than 100

*3. How many professors are teaching in your CAM program(s)?*

❑ 1-10

❑ 11-20

❑ 21-30

❑ 31-40

❑ 41-50

❑ more than 50

*4. How many of your professors have the following diplomas (some might have more than one diploma)?*

Graduate training in a CAM field: _____________

Other biomedical practitioner (e.g. RN, OT, PT): _______________

MD: ____________

PhD: ____________

*5. Are some professors involved in research activities?*

❑ Yes

❑ No

Comments:______________________________________________________________________________________________________________________________________

**Section 3: Describe the training offered at your CAM school**

*1. Type of CAM taught (please check all that apply):*

❑ Chiropractic

❑ Naturopathy

❑ Acupuncture/traditional Chinese medicine

❑ Massage Therapy

❑ Other(s), specify: _________________________

*2. Which CAM diploma(s) are offered at your school?*

________________________________________________________________________________________________________________________________________________

*3. How many hours are necessary to obtain this/these diploma(s)?*

*________________________________________________________________________________________________________________________________________________*

*4. In your curriculum, do you offer continuing education?*

❑ Yes, specify the number of hours: ___________________

Specify the teaching method (check all that apply):

❑Lectures

❑Readings

❑Invited guests

❑Case studies

❑Electives

❑Research project

❑Internships

❑ No

*5. In your curriculum, do you offer research courses?*

❑ Yes, specify the number of hours: __________________

Specify the teaching method (check all that apply):

❑Lectures

❑Readings

❑Invited guests

❑Case studies

❑Electives

❑Research project

❑Internships

❑ No

*6. In your curriculum, do you cover scientific proofs about the efficacy and safety of treatments to students?*

❑ Yes, specify the number of hours: __________________

Specify the teaching method (check all that apply):

❑Lectures

❑Readings

❑Invited guests

❑Case studies

❑Electives

❑Research project

❑Internships

❑ No

*7. In your curriculum, do you cover how CAM professionals should interact with biomedical peers (e.g. nurse, rehabilitation professional, medical doctor) in their practice?*

❑ Yes, specify the number of hours: __________________

Specify the teaching method (check all that apply):

❑Lectures

❑Readings

❑Invited guests

❑Case studies

❑Electives

❑Research project

❑Internships

❑ No

*8.* Are you satisfied of the training your school provides in the following areas?

| *Continuing education* | ❑ *Very dissatisfied*  ❑ *Dissatisfied*  ❑ *Neither satisfied not dissatisfied*  ❑ *Satisfied*  ❑ *Very satisfied* | Comments: |
| --- | --- | --- |
| *Research* | ❑ *Very dissatisfied*  ❑ *Dissatisfied*  ❑ *Neither satisfied not dissatisfied*  ❑ *Satisfied*  ❑ *Very satisfied* | Comments: |
| *Scientific proofs* | ❑ *Very dissatisfied*  ❑ *Dissatisfied*  ❑ *Neither satisfied not dissatisfied*  ❑ *Satisfied*  ❑ *Very satisfied* | Comments: |
| *Interactions with biomedical peers* | ❑ *Very dissatisfied*  ❑ *Dissatisfied*  ❑ *Neither satisfied not dissatisfied*  ❑ *Satisfied*  ❑ *Very satisfied* | Comments: |

*9. Do you expect changes in the next 2-5 years to be made to the curriculum concerning these areas?*

❑ Yes, specify:___________________________________________________________ ________________________________________________________________________❑ No

Comments:______________________________________________________________________________________________________________________________________

*10. Which improvements do you think should be made with respect to the curriculum in* those areas?

________________________________________________________________________________________________________________________________________________

________________________________________________________________________

*11. Do you think students would support such changes?*

❑ Very unlikely

❑ Unlikely

❑ Neither likely nor unlikely

❑ Likely

❑ Very likely

Comments:______________________________________________________________________________________________________________________________________

*12. Do you think the administration of the school would support such changes?*

❑ Very unlikely

❑ Unlikely

❑ Neither likely nor unlikely

❑ Likely

❑ Very likely

Comments:______________________________________________________________________________________________________________________________________

*13. What are the barriers/challenges in making those changes to the curriculum?*

________________________________________________________________________________________________________________________________________________

________________________________________________________________________

*14. Do you have any other comments to add regarding this survey?*

________________________________________________________________________________________________________________________________________________

________________________________________________________________________

**Thank you very much for your participation to this study**
